# Paid time off and cardiovascular disease events: the Health and Retirement Study, 2010–2022

**DOI:** 10.1093/haschl/qxag134

**Published:** 2026-05-29

**Authors:** Samuel L Swift, Mussammat Snigdha Sowrin, Tali Elfassy, Adina Zeki Al Hazzouri, Barbara N Harding

**Affiliations:** College of Population Health, Health Sciences Center, University of New Mexico, Albuquerque, NM 87131, United States; College of Population Health, Health Sciences Center, University of New Mexico, Albuquerque, NM 87131, United States; Katz Family Division of Nephrology and Hypertension, University of Miami Miller School of Medicine, Miami, FL 33136, United States; Columbia Mailman School of Public Health, Department of Epidemiology, NewYork, NY 10032, United States; College of Population Health, Health Sciences Center, University of New Mexico, Albuquerque, NM 87131, United States

**Keywords:** paid time off, paid sick leave, paid vacation time, myocardial infarction, stroke, cardiovascular disease

## Abstract

**Background:**

The United States does not guarantee universal access to employer-based paid time off. Several studies demonstrate the relationship between workplace benefits and cardiovascular disease (CVD) risk. The objective of our analysis was to evaluate the relationship between access to employer-based paid time off and the risk of incident CVD events in a sample of working older adults in the United States.

**Methods:**

Our sample consisted of 5604 persons aged 50 years and older from the 2010 and 2016 longitudinal prospective cohorts of the Health and Retirement Study, a nationwide US sample. Our exposure was whether participants had access to employer-based paid time-off benefits at baseline. Our outcome was incident CVD events, including myocardial infarctions, strokes, and transient ischemic attacks. We compared rates of CVD events among persons with and without benefits.

**Results:**

81.5% of our sample had paid time-off benefits. In adjusted models, persons with access to paid time-off benefits had a 32% lower risk of a CVD event over 6–14 years (mean: 7.5 years) of follow-up (hazard ratio: 0.68; 95% CI: 0.49, 0.94).

**Conclusion:**

Paid time-off benefits were associated with a reduced risk of CVD events among older adults in the United States.

## Introduction

In the United States, cardiovascular disease (CVD) is a leading cause of mortality.^1^ Several studies demonstrate the relationship between workplace conditions across the life course and CVD risk.^2-9^ Workplace conditions, including low pay,^5^ long working hours,^9^ shift work,^6^ increased workplace stress,^3^ the absence of workplace benefits such as health insurance,^7^ pensions, and lack of adequate paid time off (PTO),^2,8^ are all associated with CVD.

Unlike other comparable high-income countries, the US federal government does not universally guarantee paid sick leave^10^ or paid vacation^11^ for workers. However, there are many state- and organizational-level policies that do ensure that workers in the United States have access to these benefits. According to 2024 data, 18 US states had some laws mandating that employers provide sick leave for some employees, and although an estimated 78% of private sector workers have access to paid sick leave, a large portion of workers do not.^12^ In addition to not having guaranteed paid sick leave, workers in the US also have less PTO for vacations than comparable high-income nations.^11^ Further, females and ethnic minority populations are less likely to receive either of these PTO benefits^13^ in comparison with non-Hispanic White male workers. Access to paid vacations has been associated with reductions in heart rate^14^ and reduced metabolic syndrome risk^15^ in some small samples. Additionally, other, larger studies show workers with access to paid sick leave increase their utilization of preventive health care,^16-18^ including early detection and management of cardiovascular risk.^18^ Paid sick leave has also been associated with reductions in psychosocial stress,^19,20^ which is a major contributor to increased CVD risk.^21^ Given this literature, it is likely that access to PTO benefits may increase the use of preventive health care, reduce psychosocial stress, and thus prevent CVD events in the United States.

To our knowledge, there is no previous longitudinal analysis of cohort study data in the United States analyzing the relationship between PTO and subsequent risk of CVD. One analysis of the National Health Interview Survey demonstrated that workers who had access to employment-based paid sick leave had a reduced risk of all-cause and CVD-specific mortality.^2^ Our study aims to build on this research by examining the association between paid vacation and paid sick leave with risk of incident CVD events, specifically the risk of stroke and myocardial infarction (MI). We examined CVD risk associated with both of these benefits combined and individually, since they are often utilized interchangeably. The objective of our analysis was to evaluate the relationship between access to employer-provided PTO and subsequent risk of MIs and strokes in a sample of US adults aged 50 years and older in the United States.

## Data and methods

### Study sample: the Health and Retirement Study

The Health and Retirement Study (HRS) is an ongoing, population-based prospective cohort study, which has recruited over 40 000 persons aged 50 years and older from all 50 US states since 1992. Other details of the study procedures are described elsewhere.^22^ In brief, the HRS uses a replenishing recruitment design, by which every 6 years a new cohort of older adults is recruited into the study. Our present analysis included participants who entered into the 2010 and 2016 cohorts, which are called the “mid baby boomers” (persons born between 1954 and 1959) and the “late baby boomers” (persons born between 1960 and 1965), respectively. We excluded persons who reported MIs, strokes, or transient ischemic attacks (TIAs) prior to their entry to the cohort. Our initial sample consisted of 10 992 persons who joined the HRS in these years. We removed 5013 persons who were not asked to answer questions related to PTO in 2010 or 2016. Persons who reported marginal or no employment were not asked these questions. Marginal employment was classified as working fewer than 10 hours a week or 20 weeks per year in the 2 years prior to the interview wave. We removed 143 additional persons with missing or incorrectly reported (negative or longer than study duration) follow-up time, and 232 more participants with CVD events prior to the start of our study, arriving at an analytical sample of 5604 persons (Figure S1).

### Exposure: baseline employment-based PTO

For our main analysis, we combined 3 questions related to employment-based PTO, which were “How many days of paid sick leave at full pay do you earn each year?”, “How many weeks of paid vacation do you earn each year?”, and “How many weeks of paid time off do get each year?” These questions were measured at baseline, which was 2010 and 2016 for our 2 respective cohorts. Participants were classified as having either workplace benefit if they answered a number of days/weeks greater than zero to any of these 3 questions. In our sample, all participants who answered a non-zero number to the PTO question reported some paid sick leave or paid vacation as well. Participants were classified as not having any of these benefits if they answered “no” to all of these questions or “no” to any question with missing values for the other questions. If participants had missing data for all 3 questions, they were classified as missing exposure status. In order to examine the association between quantitative number of days of PTO and our outcome, we evaluated the relationship between tercile of number of days of PTO and incident CVD outcomes. We determined these categories by adding together the days of reported paid sick leave and paid vacation, and calculating terciles of that distribution. For this analysis, we excluded potential miscoded extreme values of number of days per year, limiting our number of days allotted per year to less than or equal to 100. We then classified participants into these groups based on the whole number of days that a person had off per year which were as follows: zero days of PTO per year, 1 to 8 days of PTO, and greater than 8 days of PTO per year. We chose this approach over simply modeling the continuous number of days off per year, as a large number of persons in our sample (19%) had zero days off per year, while others had very high numbers of days off per year, preventing us from being able to detect meaningful associations associated with single-day increments of increased PTO per year. This approach may have the added benefit of identifying thresholds at which the association may become relevant.

### Outcome: CVD events

For our main outcome, we combined the 2 major CVD events that have event time data reported within the HRS, which are self-reported MI and self-reported stroke or TIA. To be considered an incident CVD event, a participant answered “yes” to the following questions for heart attack and stroke: “[Since the previous interview/In the last two years], have you had a heart attack or myocardial infarction”, and “Has a doctor ever told you that you have had a stroke?” OR “Since we last talked to you [in the previous interview] has a doctor told you that you have had a stroke?” For the stroke question, there was an additional option of answering “yes, possible stroke or transient ischemic attack.” Participants who answered “yes” to any of these questions in the year they entered the cohort or answered “yes” to a separate question about ever having a heart attack before their year of cohort entry were considered to have prevalent CVD events and excluded from the sample, as explained previously. For participants whose events were fatal, these questions were asked of a proxy respondent.

### Follow-up time

At each wave, participants were asked about the year and month of their most recent event. For participants with complete data on the year and month of their interview and the year and month of their most recent event, follow-up time was calculated as the difference between their first interview date at entry to the cohort and the date of their most recent event. For participants who reported both a stroke and an MI, the follow-up time ended at whichever event came first, and the second event was not included in the analysis. For participants missing month of recent event but not year, month was coded as June (year midpoint). For participants who reported a new event but not a year or month, follow-up time was recorded as the midpoint between their initial interview date and the interview date of the wave in which they reported the event. These 2 missing data situations only applied to a small number (*n* = 20) of participants in the final analytical sample, as the rest had complete data on follow-up time. For participants who did not have either of these events, follow-up time was calculated as the difference between their first interview date at entry to the cohort and last interview date, or last date known alive. All person-time was measured in weeks.

### Covariates

We sequentially included the following variables as confounders in our models. All confounders were measured at baseline entry into the cohort, which was either 2010 or 2016. We defined age, which was calculated using birth date, in continuous years. We defined self-reported race/ethnicity using the categories “non-Hispanic White/Caucasian,” “non-Hispanic Black/African American,” “Hispanic (of any race),” and “Other.” We included self-reported male or female gender. We included year of cohort entry as a covariate, defined as 2010 or 2016 entry. We included level of education, operationalized as “high school education or less” vs “some college or more.” It is important to note that participants who still reported having more than marginal employment for pay, were asked these questions about their employment status. We therefore defined employment status using the categories “employed,” “other,” “retired, but working part time,” and “temporarily laid off.” We defined health insurance status using categories of “any health insurance” vs “no health insurance.” We defined baseline income measured in dollar amount income from all income sources, described elsewhere.^23^ We defined body mass index in continuous units at baseline using self-reported height and weight. We defined dichotomous physical activity as self-reported “vigorous” or “moderate” physical activity “more than once a week” vs “once per week or less.” We define smoking status using the self-reported categories “never smoker,” former smoker,” and “current smoker.” Hypertension was defined using a dichotomous “yes/no” answer to a question that was worded as follows: “Has a doctor ever told you that you have high blood pressure or hypertension?” Finally, diabetes was defined using a dichotomous “yes/no” answer to a question that was worded as follows: “Has a doctor ever told you that you have diabetes or high blood sugar?”

### Statistical analysis

We first present descriptive statistics of our sample, stratified across categories of workplace PTO, using *t*-tests to test differences across our exposure for continuous variables and χ^2^ tests to test differences across our exposure for categorical variables. Next, we present survival curves using the Kaplan-Meier method, stratified across categories of workplace PTO. We also present unadjusted incidence density rates, rate ratios, and rate differences (RDs), with PTO as our exposure and CVD events as our outcome, using Rothman's formulas to estimate CIs for rate ratios and RDs.^24^ We used 3 Cox proportional hazards models to estimate multivariable-adjusted associations between PTO and CVD events, sequentially adding more baseline covariates with each model. Our first model was adjusted for age, race/ethnicity, gender, and year of cohort entry. Our second model was additionally adjusted for educational level, employment status, income, and health insurance status; and our final model was additionally adjusted for hypertension, diabetes, smoking status, physical activity, and body mass index. Finally, we evaluated the relationship between tercile of number of days of PTO and incident CVD outcomes, adjusting for all covariates in our final model. Due to our sub-setting of the sample, that the PTO questions were administered to participants with different probabilities of selection than their survey weights reflect, and the potential for overinflating variance, we did not use HRS study weights in our main analysis models nor did we interpret our results as nationally representative. This approach is supported by recent recommendations for statistical survey-weighted research.^25,26^ Missing data on covariates were handled using listwise deletion in all models.

### Secondary and sensitivity analyses

We performed several sensitivity analyses to confirm the robustness of our results and further evaluate our research question. First, we evaluated a 4-level categorization of workplace benefits including the following 4 categories: (1) participants reporting only PTO (but not paid vacation), (2) reporting only paid vacation (but not paid sick leave), (3) participants reporting both benefits, and (4) participants with no benefits, which was the reference group, adjusting for all model 3 variables. For all stratified sensitivity analyses we did not adjust for employment status, due to the small number of people in strata besides “employed,” which caused estimation problems in our models. Second, we stratified our results by year of cohort entry (2010 and 2016) to see if our findings were consistent across the HRS cohort, adjusting for all model 3 variables, with the exception of employment. Third, we analyzed the relationship between PTO and each CVD outcome (MI and stroke) separately, repeating our analysis with all model 3 covariates, with the exception of employment. Fourth, we conducted an additional sensitivity analysis repeating our main analysis with model 3 covariates, but restricting our sample to only those reporting full-time employment at baseline. Fifth, we conducted an analysis examining quintiles of days of PTO in an attempt to further quantify the number of days of PTO that may be meaningfully associated with our outcome. Finally, to understand whether access to PTO can reduce inequalities in CVD across strata of race/ethnicity and gender, we specified models with interaction terms between PTO and gender and race/ethnicity.

## Results

Of the 5604 persons in our sample, 1046 (18.5%) had no PTO benefits and 4558 (81.5%) had PTO benefits at baseline. The mean age of persons with PTO benefits was 52.9 years compared with 52.6 years for persons without benefits. The majority of the sample was female (54.5%), and a smaller proportion of persons with PTO benefits were female (53.1%) than those who did not have PTO benefits (60.4%). Table 1 includes other descriptive characteristics of the sample, displayed across strata of our exposure of PTO benefits.

**Table 1:** Characteristics of analytical sample by workplace paid time-off benefits: the Health and Retirement Study, 2010 to 2022.

	Overall (N = 5604)	Has any paid time-off benefits (*n* = 4558)	No paid time-off benefits (*n* = 1046)	*P* value^a^
Type of benefits, No. (%)				
Has paid sick leave only	230 (4.1)	230 (5.2)	0 (0)	N/A
Has vacation time only	1537 (27.6)	1537 (34.0)	0 (0)	
Has both benefits	2758 (49.5)	2758 (61.0)	0 (0)	
No benefits	1046 (18.8)	0 (0)	1046 (100)	
Age at baseline, mean (SD), y	52.8 (4.4)	52.9 (4.2)	52.6 (5.0)	.024
Race, No. (%)				<.001
Non-Hispanic White	2525 (45.1)	2148 (47.2)	377 (36.1)	
Non-Hispanic Black	1529 (27.3)	1252 (27.5)	277 (26.6)	
Non-Hispanic Other	386 (6.9)	309 (6.8)	77 (7.4)	
Hispanic	1157 (20.7)	845 (18.6)	312 (29.9)	
Female gender, (ref = male), No. (%)	3052 (54.5)	2420 (53.1)	632 (60.4)	<.001
Study entry, No. (%)				.611
2016 cohort	2351 (42.0)	1920 (42.1)	431 (41.2)	
2010 cohort	3253 (58.0)	2638 (57.9)	615 (58.8)	
Some college or more (ref = high school or less), No. (%)	2653 (47.3)	2260 (49.6)	393 (37.6)	<.001
Household income, mean (SD), USD	$50 924.7 ($49 786.3)	$56 708.4 ($50 124.4)	$25 743.6 ($39 413.7)	<.001
Employment status, No. (%)				<.001
Employed	5387 (96.1)	4483 (98.4)	904 (86.4)	
Other but working part time	44 (0.8)	16 (0.4)	28 (2.7)	
Retired but working part time	28 (0.5)	6 (0.1)	22 (2.1)	
Temporary unemployment	144 (2.6)	52 (1.1)	92 (8.8)	
Has health insurance (ref = uninsured), No. (%)	4116 (74.4)	3658 (81.1)	458 (44.8)	<.001
Has hypertension (ref = no hypertension), No. (%)	2290 (40.9)	1898 (41.6)	392 (37.5)	.015
Has diabetes (ref = no diabetes), No. (%)	887 (15.8)	726 (15.9)	161 (15.4)	.708
Smoking status, No. (%)				.014
Never	2823 (50.4)	2318 (50.9)	505 (48.3)	
Former	1685 (30.1)	1382 (30.3)	303 (29.0)	
Current	1094 (19.5)	856 (18.8)	238 (22.8)	
Body mass index, mean (SD), kg/m^2^	29.8 (6.3)	29.9 (6.2)	29.4 (6.4)	.017
Vigorous or moderate physical activity more than once per week (ref = once per week or less), No. (%)	3456 (61.7)	2810 (61.7)	646 (61.8)	.961

N = 5604.

Abbreviations: N/A, not available; ref, reference; USD, US dollars.

^a^ *P* values obtained using χ^2^ tests for categorical values and *t*-tests for continuous variables.

**Figure 1:**
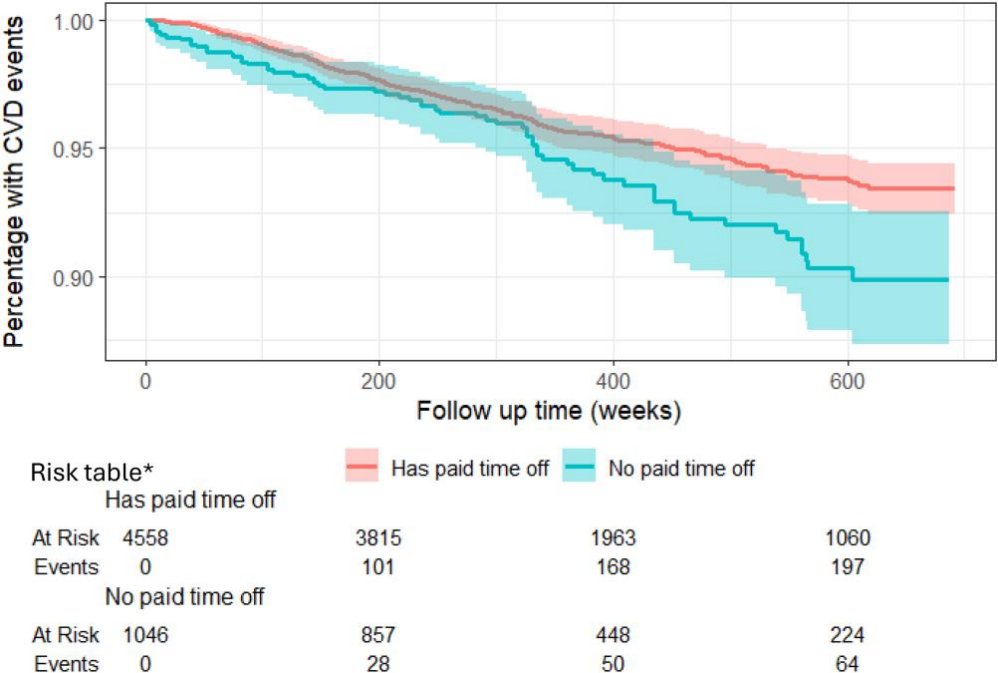
Unadjusted Kaplan-Meier survival curve comparing CVD events across persons with and without workplace paid time off, the Health and Retirement Study, 2010-2022 (*n* = 5604). *Numbers in the risk table refer to numbers at risk and number of events in each group at 0, 200, 400, and 600 weeks of follow-up. Abbreviation: CVD, cardiovascular disease events defined by stroke and myocardial infarctions.

There were 265 CVD events over follow-up in our study, with 200 (incidence rate: 11.2 cases per 100 000 person-weeks) among persons with PTO benefits and 65 (incidence rate: 16.1 cases per 100 000 person-weeks) among persons with no PTO benefits (Table 2, Figure 1). In our unadjusted analysis, persons with PTO had 4.8 fewer events per 100 000 person-weeks (RD: −4.8; 95% CI: −9.0, 0.0) compared with persons without the PTO (Table 2).

**Table 2:** Rates of cardiovascular disease events by paid time off: unadjusted incidence rates, rate ratios, and rate differences: the Health and Retirement Study, 2010–2022.

	No. of CVD events	Person-time, wk	Incidence rate^a^	Rate ratio (95% CI)	Rate difference^a^ (95% CI)
Has paid time off	200	1 779 294.1	11.2	**0.70** **(0.52, 0.93)**	**−4.8** **(−9.0, −0.0)**
Does not have paid time off	65	404 855.6	16.1	Ref	Ref

Bold indicates statistically significant findings. Cardiovascular disease events are defined by stroke, transient ischemic attacks, and myocardial infarctions.

Abbreviations: CVD, cardiovascular disease; Ref, reference.

^a^Incidence rate and risk difference are cases per 100 000 person-weeks.

Table 3 shows the multivariable-adjusted Cox models. In model 1, after adjusting for age, race/ethnicity, gender, and year of cohort entry, the persons with PTO had a 34% lower risk of CVD events compared with persons without PTO (model 1: hazard ratio [HR]: 0.66; 95% CI: 0.50, 0.88). This association was similar in our final model, with persons with PTO having a 32% lower risk of CVD events than person without PTO (HR: 0.68; 95% CI: 0.49, 0.94).

**Table 3:** Associations between workplace paid time off and subsequent cardiovascular disease events using multivariable-adjusted Cox proportional hazards models: the Health and Retirement Study, 2010–2022.

	HR (95% CI)		
	Model 1^a^	Model 2^b^	Model 3^c^
Has paid time off	**0.66** **(0.50, 0.88)**	**0.72** **(0.53, 1.00)**	**0.68** **(0.49, 0.94)**
Does not have paid time off	Ref	Ref	Ref

Bolded values denote confidence intervals that do not cross one. Cardiovascular disease events are defined by stroke, transient ischemic attacks, and myocardial infarctions.

Abbreviations: HR, hazard ratio; Ref, reference.

^a^Model 1 was adjusted for age, race/ ethnicity, gender, and year of cohort entry.

^b^Model 2 was additionally adjusted for educational level, employment status, income, and health insurance status.

^c^Model 3 was additionally adjusted for hypertension, diabetes, smoking status, physical activity, and body mass index.

Table 4 shows our analysis examining the relationship between tercile of days of PTO and CVD events. Compared with persons with no PTO, persons with more than 8 days of PTO per year had a 45% reduced risk of CVD events (HR: 0.55; 95% CI: 0.37, 0.82), and persons with 1 to 8 days of PTO per year had a 35% reduced risk of CVD events (HR: 0.65; 95% CI: 0.46, 0.92).

**Table 4:** Associations between tercile of number of days of workplace paid time off and subsequent cardiovascular disease events: the Health and Retirement Study, 2010–2022.

	HR (95% CI)
More than 8 days of PTO per year	**0.55** **(0.37, 0.82)**
1 to 8 days of PTO per year	**0.65** **(0.46, 0.92)**
0 days of PTO per year	Reference

Bold indicates statistically significant findings*. n* = 4904. This model was adjusted for age, race/ethnicity, gender, year of cohort entry, educational level, employment status, income, health insurance status, hypertension, diabetes, smoking status, physical activity, and body mass index. Cardiovascular disease events are defined by stroke, transient ischemic attacks, and myocardial infarctions. This model is restricted to participants reporting 100 or less days of PTO per year.

Abbreviations: HR, hazard ratio; PTO paid time off.

Our sensitivity analysis using a 4-level construct of PTO benefits was consistent with our main analysis in magnitude and direction of associations (Table S1). Our sensitivity analysis stratifying results by year of cohort entry (2010 vs 2016) was consistent in directionality with our main analysis; however, the estimate was much smaller and not statistically significant for the 2016 cohort (Table S2). Table S3 shows multivariable-adjusted Cox proportional hazards models showing associations between workplace PTO and subsequent MI and strokes separately. While not significant for MIs, the association for MIs, strokes, and TIAs individually was similar to our main analysis in magnitude and direction of association. Table S4 shows our analysis restricting our sample to persons reporting full employment at baseline. The results of this analysis were significant, and similar in magnitude and direction to our main analysis. Table S5 shows that, compared with persons with no PTO, there were significant associations with a reduced risk of CVD events in all quintiles of days of PTO per year except for 3 to 6 days off per year (the third quintile). Finally, we did not find significant interactions between race/ethnicity and sex in models with interaction terms (analysis not shown).

## Discussion

We found that persons who had any PTO benefits through their work had a 32% reduced risk of experiencing either an MI, stroke, or TIA over 6 to 12 years of follow-up, in models adjusted for demographic, socioeconomic, and lifestyle-related covariates. This association was consistent across several sensitivity analyses, and we found a dose–response relationship when examining terciles of number of days of PTO. While subject to selection biases due to the observational design, in context with other literature on this topic, these findings have important implications for public health practice and advocacy for these benefits in the United States.

There are several mechanistic pathways through which access to PTO may influence CVD. In other observational studies, paid sick leave has been associated with reductions in psychological distress,^20^ reductions in depression and anxiety,^19,20^ and increased use of preventive care^16,17-18^. In contrast, another observational analysis found that workers without access to paid sick leave are more likely to forgo necessary medical care, both for themselves and for family members.^27^ While fewer longitudinal studies have looked at the relationship between vacation time and CVD events, there is evidence that access to paid vacation time, such as paid sick leave, is associated with reduced stress.^14^ In turn, poor mental health is associated with increased risk of CVD events,^28^ while increased use of preventive care is associated with reduced risk of CVD events.^29^

Previous research from our team demonstrated that state-level paid sick leave mandates were associated with a reduction in CVD mortality in the northeastern region of the United States.^8^ Recent additional research has demonstrated that state-level paid sick mandates improved self-rated health among working-age women.^30^ At the organizational level, a meta-analysis found that paid sick leave is associated with several favorable benefits for employers themselves, including increased employee retention and presenteeism.^31^ Finally, another study from Canada found that paid vacation was also associated with a reduction in voluntary turnover.^32^ This finding is consistent with other research, which argues that these benefits may provide advantages for both employers and employees, including reduced turnover and increased employee productivity.^33^ Given the results of our current observational study within the greater context of other research that uses quasi-experimental causal identification strategies,^8,30^ we believe that there is scientific evidence demonstrating that these benefits and the policies that ensure them may be cost-effective tools for CVD prevention and public health more broadly. Given our results in conjunction with others, we advocate for the use of existing interventions at the organizational, state, and federal levels that may improve access to these benefits and may therefore reduce the risk of CVD.

This study has several limitations. First, CVD events and time of CVD events were both self-reported, allowing for the possibility of recall bias in both of these variables. We believe this bias may be nondifferential, which commonly underestimates associations. Person-time was also self-reported, which may be subject to recall bias, especially in persons who experienced cognitive impairment as a result of a stroke or TIA, as research shows that self-reported CVD events are prone to recall bias in comparison with adjudicated CVD events.^34^ However, we believe that MIs and strokes or TIAs are major life events, and thus may be less subject to recall bias than other self-reported measures. We acknowledge that our measure of exposure was measured only once at baseline, and that this could be improved by examining length of employment and benefits or time-varying exposure over follow-up. Paid time off is a dynamic variable, and several states mandated paid sick leave during our study period, meaning that some workers without PTO may have gotten it during our study time period, while others may have lost employment and thus lost this benefit. However, given that the HRS is among older adults and many retire over follow-up, this is difficult to examine in our sample. To our knowledge, few longitudinal cohorts in the United States take repeated measures of these types of benefits starting earlier in the life course, which would improve the measurement of this exposure. Our analysis on quintiles of days of PTO was not consistent with our analysis of terciles of days of PTO, weakening the evidence of a dose–response relationship. A key limitation is that, unlike other studies on this topic with a clear causal identification strategy,^8,30^ this analysis is descriptive, lacking exogeneity in the exposure, and therefore subject to selection bias wherein persons who self-select into jobs with PTO may be healthier than persons who do not self-select into these jobs. While we attempted to address this bias by adjusting for several key covariates, our results remain purely observational. We recommend that our results be considered in the context of the results of research with stronger designs, which found results consistent with ours.^8,30^ We do not claim that our results are nationally representative, and in fact, the number of adults in our sample with PTO was higher (81%) than the number of persons in the United States with these benefits (60%–66%) during a comparable time period, as reported by data from the Bureau of Labor Statistics.^35^ Our variable did not include all types of paid leave using broader definitions of PTO,^12^ including paid family medical leave, which was not included in our analysis. We recommend that future work further evaluates different types of policies mandating PTO for employees, including paid family medical leave policies, which were not included in this analysis.

Our study has notable strengths as well. To our knowledge, this is the first analysis to examine the relationship between PTO and CVD events in a longitudinal US cohort, and we were able to examine different constructs of both our exposure (paid sick leave and vacation) and different aspects of our outcome (MIs, strokes, and TIAs). Furthermore, we demonstrated a dose–response relationship when examining tercile of number of days of PTO, although this relationship was not present when examining the more granular variable of quintiles of days of PTO. While many previous studies look at paid sick leave alone, we expanded on this research by including paid vacation in our analysis. Our results were consistent across all of our secondary and sensitivity analyses. While not all results were statistically significant, results were consistent in direction and magnitude. In supplementary sensitivity analysis, we did not find consistent differences across type of benefit, suggesting that both benefits may contribute to the prevention of CVD events. We found a much stronger relationship between benefits and CVD events in the 2010 cohort vs the 2016 cohort, which is likely due to the accumulation of more follow-up time for the 2010 cohort, resulting in more precise estimates. We found similar associations when examining strokes and TIA events separately from MIs, and the nonsignificant result for MIs is likely also attributable to statistical power.

In conclusion, we demonstrated that employer-based PTO benefits were associated with a reduction in the risk of CVD events. We recommend that future research examines more complex constructs of employer-based time-off benefits, over longer periods of the life course. Since many policies already exist for ensuring workers access to these benefits at the organizational, state, and federal levels, we recommend that the medical, scientific, and public health communities advocate for these benefits as public health interventions for CVD prevention.

## Supplementary Material

qxag134_Supplementary_Data
