# Supplementary material for: Paid time off and cardiovascular disease events: the Health and Retirement Study, 2010–2022
Source: Health Aff Sch. 2026 May 29;4(6):qxag134. doi: 10.1093/haschl/qxag134 (PMC13268761; doi:10.1093/haschl/qxag134)
Supplement: qxag134_Supplementary_Data [file qxag134_supplementary_data.zip › Supplementary_Table S1-S5.docx]

**Supplementary Table 1.** Associations between workplace paid vacation time, paid sick leave, and subsequent cardiovascular disease eventsꝉ using multivariable adjusted Cox proportional hazards models, The Health and Retirement Study 2010-2022

|  | HR (95% CI) |
| --- | --- |
| Has both paid sick leave and paid vacation time (n=2,758) | 0.72  (0.51, 1.01) |
| Has paid sick leave only (n=230) | 0.57  (0.27, 1.20) |
| Has paid vacation only (n=1,537) | **0.65**  **(0.49, 0.95)** |
| Does not have paid time off (n=1,046) | ref |

HR= Hazard Ratio

*This model is adjusted for age, race/ ethnicity, gender, year of cohort entry, education level, employment status, income, health insurance status, hypertension, smoking status, physical activity, and body mass index

ꝉCardiovascular disease events defined by stroke, transient ischemic attacks, and myocardial infarctions

ꝉCardiovascular disease events defined by stroke, transient ischemic attacks, and myocardial infarctions

**Supplementary Table 2.** Associations between workplace paid time off and subsequent cardiovascular disease eventsꝉ using multivariable adjusted Cox proportional hazards models, stratified by year of cohort entry, The Health and Retirement Study 2010-2022

|  | 2010 cohort (n=3,260)  Mean follow up time = 9.5 years | 2016 cohort (n=2,357)**  Mean follow up time = 4.7 years |
| --- | --- | --- |
|  | HR (95% CI) | HR (95% CI) |
| Has paid time off | **0.62**  **(0.43, 0.88)** | 0.89  (0.44, 1.78) |
| Does not have paid time off | ref | ref |

HR= Hazard Ratio

*All models are adjusted for age, race/ ethnicity, gender, education level, income, health insurance status, hypertension, diabetes, smoking status, physical activity, and body mass index

ꝉCardiovascular disease events defined by stroke, transient ischemic attacks, and myocardial infarctions

**Supplementary Table 3.** Associations between workplace paid time off and subsequent myocardial infarctions, and strokes separately using multivariable adjusted Cox proportional hazards models, The Health and Retirement Study 2010-2022

|  | Myocardial Infarctions* | Strokes and TIAs** |
| --- | --- | --- |
|  | HR (95% CI) | HR (95% CI) |
| Has paid time off | 0.69  (0.43, 1.12) | **0.64**  **(0.42, 0.98)** |
| Does not have paid time off | ref | ref |

*****Stroke free during follow up sample for myocardial infarction analysis N=5,457, events= 108

******Myocardial infarction free during follow up sample for stroke analysis N=5,492, events= 142

TIA= Transient Ischemic Attacks

***All models are adjusted for age, race/ ethnicity, gender, year of cohort entry, education level, income, health insurance status, hypertension, diabetes, smoking status, physical activity, and body mass index

**Supplementary Table 4.** Associations between workplace paid time off and subsequent cardiovascular disease eventsꝉ using multivariable adjusted Cox proportional hazards models, among a sample restricted to only persons working full time (n=5,184), The Health and Retirement Study 2010-2022

|  | HR (95% CI) |
| --- | --- |
| Has paid time off | **0.69**  **(0.49, 0.97)** |
| Does not have paid time off | ref |

HR= Hazard Ratio

*This model is adjusted for age, race/ ethnicity, gender, year of cohort entry, education level, employment status, income, health insurance status, hypertension, diabetes, smoking status, physical activity, and body mass index

ꝉCardiovascular disease events defined by stroke, transient ischemic attacks, and myocardial infarctions

**Supplementary Table 5.** Associations between quintile of number of days of workplace paid time off and subsequent cardiovascular disease events, The Health and Retirement Study, 2010-2022 (N=4,678)

|  | HR (95% CI) |
| --- | --- |
| Greater than 12 days of paid time off per year | **0.56**  **(0.36, 0.87)** |
| 7 to 12 days off per year | **0.61**  **(0.40, 0.94)** |
| 3 to 6 days off per year | 0.80  (0.53, 1.19) |
| 1 to 2 days off per year | **0.53**  **(0.34, 0.81)** |
| 0 days of paid time off per year | Reference |

*This model is adjusted for age, race/ ethnicity, gender, year of cohort entry, education level, employment status, income, health insurance status, hypertension, diabetes, smoking status, physical activity, and body mass index

ꝉCardiovascular disease events defined by stroke, transient ischemic attacks, and myocardial infarctions

**This model is restricted to participants reporting 100 or less days of PTO per year
