# Supplementary figures and images for: Paid time off and cardiovascular disease events: the Health and Retirement Study, 2010–2022
Source: Health Aff Sch. 2026 May 29;4(6):qxag134. doi: 10.1093/haschl/qxag134 (PMC13268761; doi:10.1093/haschl/qxag134)

**Supplementary Figure 1.** Study Sample Flow Diagram, Health and Retirement Study, 2010-2022

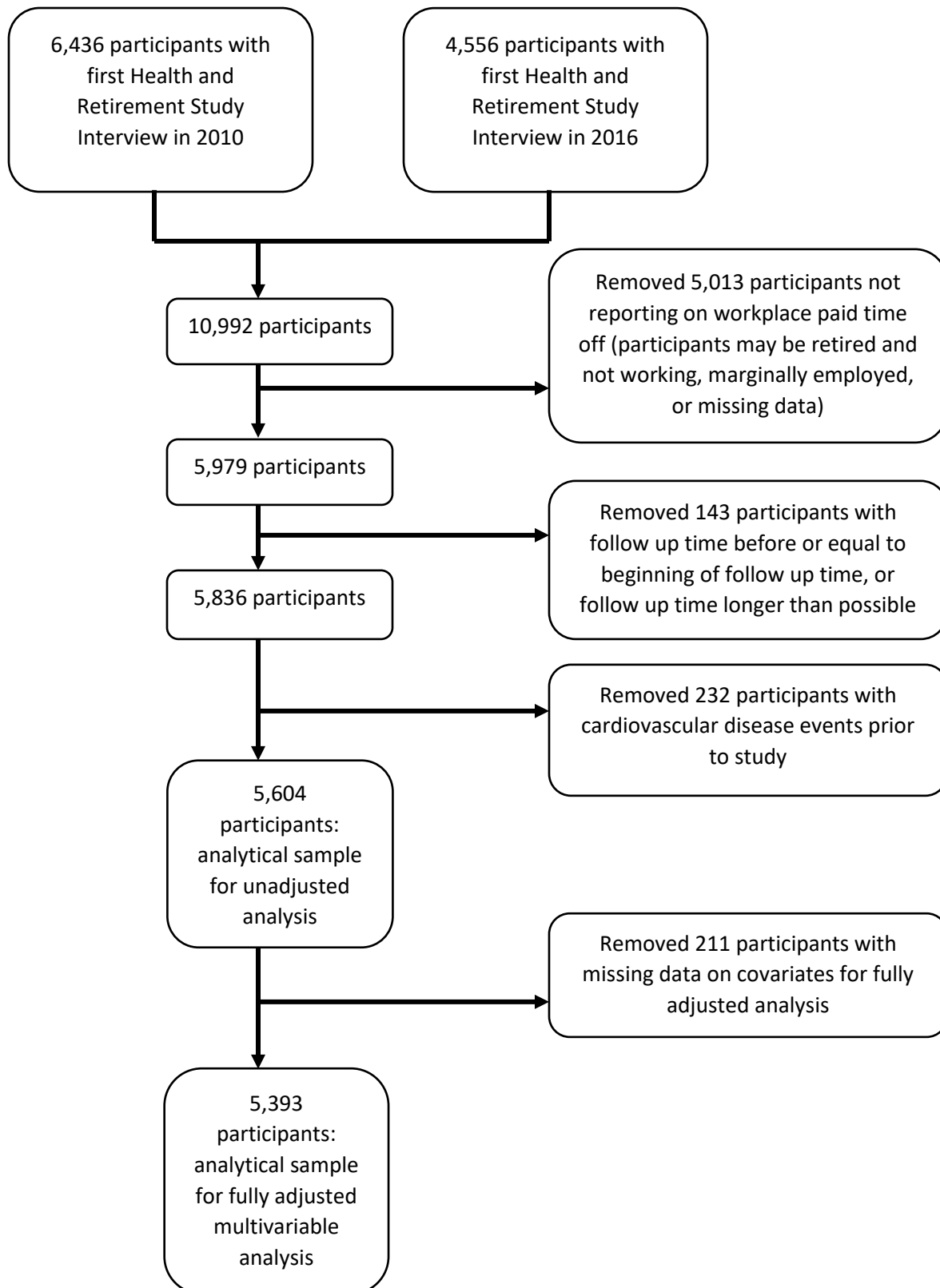

Supplement: qxag134_Supplementary_Data [file qxag134_supplementary_data.zip › Supplementary Figure 1.pdf]
